# Development of an Iranian Clinical Guideline on Perioperative Exercise Therapy for Metabolic and Bariatric Surgery: A Delphi Consensus Study Using the AGREE II Framework

**DOI:** 10.5812/ijem-166557

**Published:** 2026-05-25

**Authors:** Parisa Nejati, Ali Kabir, Ali Mazaherinezhad, Haleh Dadgostar, Mohammad Kermansaravi, Lida Nejati

**Affiliations:** 1 Department of Sports and Exercise Medicine, Hazrat-e-Rasool General Hospital, School of Medicine, Iran University of Medical Sciences, Tehran, Iran; 2 Artificial Intelligence in Health Research Center, Iran University of Medical Sciences, Tehran, Iran; 3 Minimally Invasive Surgery Research Center, Iran University of Medical Sciences, Tehran, Iran; 4 Division of Minimally Invasive and Bariatric Surgery, Department of Surgery, Minimally Invasive Surgery Reasearch Center, Hazrat-E Fatemeh Hospital, School of Medicine, Iran University of Medical Sciences, Tehran, Iran; 5 Office of Executive Dean for Research, University of Miami, Miller School of Medicine, Florida, USA

**Keywords:** Exercise Therapy, Obesity, Metabolic And Bariatric Surgery, Physical Activity

## Abstract

**Context:**

This guidance aims to advance current understanding of the benefits of exercise therapy and to present an exercise protocol for the preoperative and postoperative periods surrounding metabolic and bariatric surgery (MBS).

**Evidence Acquisition:**

A panel of experts conducted a comprehensive review of the existing literature in databases such as PubMed, Scopus, and Web of Science to gather evidence. Studies involving exercise interventions before and/or after metabolic and bariatric surgery were included. A three-round Delphi process and the AGREE (Appraisal of Guidelines for Research and Evaluation) Checklist were used to facilitate the development of the practice approach. Selected clinical questions were addressed through a consensus-based protocol, with 94% agreement on the final 16 recommendations.

**Results:**

A total of 16 clinical recommendations were formulated. The benefits of incorporating exercise therapy, as well as the optimal timing and specific protocols for exercise before and after MBS, are highlighted. The recommendations include initiating structured exercise at least three months before surgery and implementing combined aerobic and resistance exercise after surgery. These recommendations emphasize the importance of exercise therapy in improving physical function and metabolic conditions, promoting weight and fat loss, and preventing weight regain.

**Conclusions:**

This guidance draws on the latest scientific evidence. Providing clinicians and patients with obesity with an evidence-based approach to practice could help improve quality of life and reduce the burden on society and patients' families. Candidates for MBS are recommended to initiate a structured exercise program at least three months before surgery and to continue long-term participation in combined aerobic and resistance exercise after MBS to optimize metabolic health and body composition outcomes.

## 1. Context

Obesity is a public health issue with increasing prevalence worldwide (1-6). The prevalence of obesity nearly tripled from 1975 to 2016, with more than 1.9 billion adults aged 18 and older being overweight and over 650 million classified as obese in 2016 (7). The prevalence of obesity and overweight among children is also rising (1, 7). Recent national and regional data also highlight the increasing burden of severe obesity in Iranian adults, particularly in urban populations (8, 9).

Obesity is a globally prevalent disease and a risk factor for many conditions, including type 2 diabetes mellitus (T2D), cardiovascular diseases (CVD), arterial hypertension (HTN), dyslipidemia (DLP), osteoarthritis (OA), polycystic ovary syndrome (PCOS), and cancers (10). Each year, 2.8 million people die due to obesity and overweight (10). Individuals with obesity or overweight typically experience a lower quality of life (11).

Regular physical activity positively affects the reduction of the risk of obesity and overweight. However, one in four adults do not engage in the minimum recommended amount of physical activity (12). Evidence indicates that the prevalence of physical inactivity in Iran is higher than the global average, reaching between 30 and 70 percent (13, 14).

Among approaches to obesity control, metabolic and bariatric surgery (MBS) is the most effective, durable, and efficient method for improving obesity-associated medical problems, such as T2D (15). Although the number of MBS procedures has increased in recent years, studies show that physical activity levels among individuals after MBS remain low. Specifically, in 90 percent of those who have undergone MBS, physical activity levels are still insufficient, and in some patients, the amount of physical activity has even decreased compared with the period before surgery (16, 17). Therefore, in addition to surgery, it is crucial to encourage individuals to engage in physical activity and exercise and to provide the necessary conditions to increase physical activity levels before and after MBS. This underscores the importance of a standardized, credible clinical guideline or exercise protocol tailored for individuals who are candidates for MBS.

## 2. Evidence Acquisition

This clinical practice guideline, entitled “Exercise therapy in metabolic and bariatric surgery,” was developed to advise Iranian individuals with obesity regarding exercise before and after MBS. It is a joint product of the Sports Medicine Department and the Minimally Invasive Research Center at Iran University of Medical Sciences (IUMS).

After registration with the Office of Standardization and Development of Clinical Guidelines, a subsidiary of Iran’s Ministry of Health and Medical Education, a multidisciplinary team (MDT) was established. Specialists in sports medicine, metabolic and bariatric surgery, obesity medicine, nutrition, physical therapy, and epidemiology, with more than a decade of work experience in the field of obesity, were recruited as MDT members. They determined the scope and priorities, searched for resources, developed scenarios, analyzed the clinical benefits of the scenarios, managed consensus, and summarized the results. Execution of all stages of development was supervised by the team leader (P.N.), who had experience in developing five clinical guidelines and was certified by the Office of Standardization and Development of Clinical Guidelines, Iran’s Ministry of Health and Medical Education.

The objective was to collect evidence-based information to support clinical advice on the preventive and therapeutic effects of physical activity in patients who are candidates for MBS. Using a structured approach, issues pertinent to exercise for MBS were specified so that they could be communicated to patients with obesity and MDT members. The viewpoints and preferences of patients, as well as the experience of MDT members, were also incorporated. The key topics addressed the practical needs of patients and clinicians.

A comprehensive systematic search on exercise and bariatric surgery was conducted in the following databases, regardless of study design, comparator groups, outcomes, language, and context: PubMed (MEDLINE), Scopus, Web of Science, The Canadian Practice Guidelines InfoBase, Agency for Healthcare Research and Quality (AHRQ), National Institute for Health and Care Excellence (NICE: www.nice.org.uk), Scottish Intercollegiate Guidelines Network (SIGN: www.sign.ac.uk), New Zealand Guidelines Group (www.health.govt.nz/publications), and the National Health and Medical Research Council (NHMRC: www.nhmrc.gov.au). The search key terms were obesity, metabolic and bariatric surgery, exercise, physical activity, consensus, and clinical practice guideline. No specific guidelines on exercise therapy for patients who are candidates for MBS were identified. Some clinical guidelines related to obesity management were available, in which exercise therapy and physical activity were mentioned briefly as part of lifestyle therapy for weight loss (Appendix 1 in the Supplementary File). Therefore, to specifically cite evidence regarding physical activity in MBS, we relied on systematic reviews and clinical trials, and on expert opinion when evidence was insufficient. Included studies were peer-reviewed English publications (from January 2000 to June 2024) addressing exercise interventions in pre- and post-MBS contexts. Case reports, editorials, and non-clinical papers were excluded.

A questionnaire comprising 16 clinical scenarios/questions was developed. The clinical questions concerned the efficacy, cost-benefit, and feasibility of exercise, as well as the details of an exercise protocol (Appendix 2 in the Supplementary File). Gaps between knowledge and practice, as well as the clinical need for developing guidance, were considered in the clinical scenarios. The PICO framework was used to formulate the 16 clinical questions to guide the evidence search and develop the practice approach. In this framework, P stands for Population (adults undergoing metabolic or bariatric surgery in this study); I for Intervention (structured pre- and postoperative exercise therapy in this study); C for Comparisons (if appropriate); and O for Outcome(s) (weight or fat loss, improved recovery, physical function, or quality of life in this study).

To determine how current evidence supports the clinical questions, a broad search of resources was conducted. The existing answers to the questions, as well as the scientific evidence supporting each answer, were presented as recommendations by the MDT members.

The overall quality of evidence supporting each proposed recommendation was determined. The clinical relevance and applicability of the recommendations were also assessed. In addition, the advantages and disadvantages of acting on each recommendation were evaluated.

The recommendations were voted on using a three-round Delphi technique to gather a broad range of ideas, opinions, and evidence from experts. Additional considerations included effectiveness, safety, cost, feasibility, patients’ buy-in, affordability, accessibility, and alternative scenarios. To evaluate recommendations, supporting evidence, and expected clinical benefits, standardized forms developed by the Office of Clinical Guideline Standardization were used. Clinical scenarios were assessed based on the existing literature, considering treatment effectiveness, benefits, side effects, costs, and applicability. The localizability of the guidance, including feasibility, generalizability, and acceptance, was also evaluated. Examples of the forms are provided in Appendix 3 in the Supplementary File.

All panel members scored each item in the form from 1 to 3, with higher scores indicating greater suitability of each scenario. In addition, agreement among experts was calculated, with higher agreement (> 90%) indicating a higher degree of certainty. Items with lower agreement scores were submitted to the next Delphi round of the expert panel. Scenarios with higher scores were ultimately selected and published as the best. Finally, the wording and priority of each recommendation were agreed upon by the expert panel.

During the drafting and final editing stages of the guidance, the AGREE (Appraisal of Guidelines for Research and Evaluation) Reporting Checklist was used to ensure the document is evidence-based, clear, and implementable (18, 19). The purpose, applicability, clarity, and development process were assessed using the AGREE checklist.

This approach includes statements to assist practitioners in making decisions about appropriate physical activity for individuals with obesity. It can be applied to any candidate for MBS of any age, any gender, or with any associated medical problems. Different parts of this guideline would be suitable for use by policymakers; sports medicine specialists; obesity medicine specialists; metabolic and bariatric surgeons; general practitioners; dietitians; patients with obesity and their families; and administrators of obesity clinics. The guidance is accessible through the Office of Standardization and Development of Clinical Guidelines, the Ministry of Health and Medical Education of Iran (https://hetas.behdasht.gov.ir/). Moreover, it will be available through national obesity clinics for specialists and patients, and at all universities of medical sciences across the country. Feedback from the MDT and patients with obesity will be recorded to evaluate facilitators of and barriers to implementing this guidance.

This guidance is expected to be updated in 5 - 10 years.

The strength of evidence was determined according to the level and quality of evidence and statistical precision (20). Levels of evidence are as follows:

Level 1A: Evidence from systematic reviews or meta-analyses of randomized controlled trials (RCTs); Level 1B: Evidence from at least one RCT Level 2A: Evidence from at least one controlled study without randomization; Level 2 B: Evidence from at least one other type of quasi-experimental study; Level 3: Evidence from non-experimental descriptive studies, such as comparative studies, correlation studies, and case-control studies; Level 4: Evidence from expert committee reports or opinions or clinical experience of respected authorities, or both

The scientific strength of the recommendations is as follows:

Grade A: Based on level 1 evidence (1A, 1B); Grade B: Based on level 2 evidence; Grade C: Based on level 3 evidence; Grade D: Based on level 4 evidence

- Metabolic and bariatric surgery refers to various surgical procedures aimed at aiding weight loss and improving associated medical problems in individuals with severe obesity, usually when other weight loss methods have failed. The ICD-11 (International Classification of Diseases; 11th revision) code for bariatric surgery is Z98.84.

- Exercise therapy is a therapeutic approach that uses physical activity to improve health, manage chronic conditions, and accelerate recovery from injuries. In the ICD-11 system, the relevant code for exercise counseling as a factor influencing the state of health is Z71.82.

## 3. Results

### 3.1. Study Selection and Characteristics

A total of 355 studies were included in the evidence synthesis, comprising systematic reviews, meta-analyses, randomized controlled trials (RCTs), and observational studies relevant to exercise interventions before and after metabolic and bariatric surgery (MBS). Most studies focused on the short-term postoperative period, with limited data on long-term outcomes. The studies addressed various aspects of physical activity, including aerobic exercise, resistance training, and combined programs, and assessed outcomes related to weight loss, body composition, metabolic health, and physical function.

### 3.2. Quality of Evidence and Risk of Bias

The overall quality of evidence was assessed using a combination of methodological evaluation tools. While many studies were high-quality RCTs or meta-analyses, some recommendations relied on expert consensus because of the limited number of studies addressing specific questions. The risk of bias varied across studies, with some demonstrating moderate to high risk due to outcome reporting, blinding, or study design.

### 3.3. Evidence Mapping Table

To provide a comprehensive overview of the evidence supporting each recommendation, we developed Table 1 - Evidence Mapping Table. This table summarizes each of the 16 recommendations in terms of the number of supporting studies, study design (RCT, systematic review, observational study), risk of bias, level of evidence, and notes on gaps in evidence or reliance on expert opinion (Table 1). The table highlights areas with strong empirical support as well as recommendations for which guidance is primarily based on consensus, enabling clinicians to understand the robustness and limitations of the evidence.

**Table 1.** Evidence Mapping Table for Each Recommendation ^a>^

Rec	Clinical Topic	Study Design(s) Cited	No. of Studies	Risk of Bias (Overall)	Consistency of Findings	Evidence Level Assigned	Recommendation Grade	Rationale for Final Grade
**1**	Preoperative exercise → postoperative weight loss	RCTs + controlled studies	4	Moderate	Mostly consistent (3 positive, 1 neutral)	Level 1B	A	Multiple controlled trials with generally consistent outcomes supporting benefit.
**2**	Preoperative exercise → postoperative body fat %	Small RCTs / controlled studies	3	Moderate-High	Inconsistent	Level 4 (Panel decision)	D	Small sample sizes, inconsistent findings, indirect outcomes; expert consensus dominant.
**3**	Exercise type/intensity for weight, fat, muscle	Meta-analyses + RCTs	Multiple	Low-Moderate	Highly consistent	Level 1A	A	Strong meta-analytic evidence across obesity populations.
**4**	Exercise → cardiorespiratory fitness/function	RCTs + cohort studies	Multiple	Moderate	Consistent	Level 1A / 1B	A	Strong physiologic evidence across pre- and post-MBS populations.
**5**	Preoperative PA → postoperative PA behavior	Cohort + observational	Multiple	Moderate	Generally consistent	Level 2	B	Predictive association evidence; limited interventional data.
**6**	Need for medical screening before exercise	Guideline-based + consensus	Multiple guidelines	Low	Consistent	Level 1 (Guideline-derived)	A	Strong international guideline consensus + safety priority.
**7**	Postoperative exercise → weight/fat loss	RCTs + cohort studies	Multiple	Moderate	Consistent	Level 1B-2	A / B	RCT-supported benefit; magnitude varies across studies.
**8**	Postoperative aerobic progression (HRR-based)	Clinical trials + exercise physiology evidence	Several	Moderate	Generally consistent	Level 1B	A	Supported by structured exercise intervention trials + physiology evidence.
**9**	Postoperative resistance training prescription	RCTs + intervention trials	Several	Moderate	Consistent	Level 1B	A	Strong evidence for lean mass preservation and function.
**10**	Age-specific exercise recommendations	Expert consensus + indirect studies	Few	High	Limited	Level 4	D	Lack of direct age-stratified MBS exercise trials.
**11**	Exercise → insulin sensitivity	Meta-analyses + RCTs	Multiple	Low	Highly consistent	Level 1A	A	Strong metabolic physiology evidence.
**12**	Postoperative exercise → additional weight loss	RCTs + longitudinal studies	Several	Moderate	Consistent	Level 1B	A	Multiple controlled trials support benefit.
**13**	Exercise → prevention of weight regain	Longitudinal cohort + intervention	Several	Moderate	Consistent	Level 1B-2	A	Strong longitudinal evidence for maintenance effect.
**14**	Exercise → cardiorespiratory indices postop	RCTs + cohort studies	Several	Moderate	Consistent	Level 1B	A	Reproducible improvements across trials.
**15**	Exercise → muscle preservation postop	RCTs + physiology studies	Multiple	Moderate	Consistent	Level 1B-2	A / B	Strong physiologic rationale + intervention evidence; long-term adherence variable.
**16**	PA measurement tools after MBS	Observational + device validation	Limited	Moderate-High	Variable	Level 4	D	No gold standard; heterogeneous measurement methods.

^a^ Abbreviations: RCTS randomized controlled trials

### 3.4. Clinical Recommendations

Based on the evidence mapping and expert consensus, a total of 16 clinical recommendations were formulated. The benefits of incorporating exercise therapy, as well as the optimal timing and specific exercise protocols before and after MBS, are highlighted. The recommendations emphasize the importance of exercise in improving physical function and metabolic health, promoting weight and fat loss, and preventing weight regain. For example, patients are advised to begin a regular exercise program at least three months before MBS and to continue structured physical activity for as long as possible postoperatively. Both aerobic and resistance exercises are recommended, with specific prescriptions tailored to individual capacity and clinical conditions.

#### 3.4.1. Does Exercise Before MBS Lead to More Weight Loss After MBS?

Unfortunately, few studies have evaluated the effect of exercise in the preoperative period. Of the four high-quality studies conducted in this field, three show that exercise before MBS can lead to more significant weight loss in the postoperative period, even up to one year after surgery (18-20).

Only one study shows that exercising before surgery does not have any effect on weight after surgery (21).

Regular exercise before MBS results in greater adherence to continued physical activity in the postoperative period, which can lead to greater weight loss. Therefore, it is recommended that all patients with obesity who are candidates for MBS follow a regular exercise program for at least three months before MBS (A).

#### 3.4.2. Can Exercise Before MBS Reduce Body Fat Percentage After MBS?

Three studies have examined the effect of exercise before MBS on body fat after MBS, with two showing that the percentage of body fat does not change after three months of aerobic and strength training following surgery (20, 21). However, the other study found that in people who had performed a combination of aerobic and strength exercises for a period of three months leading up to MBS, the postoperative percentage of body fat was lower than in those who had not performed any exercise (18).

It is recommended to start a combination of aerobic and strength exercises for at least three months before MBS. The expert panel agrees that engaging in regular physical activity before surgery helps patients resume such activities sooner in the postoperative period. Thus, the amount of physical activity resulting in fat loss will be achieved over a shorter period (D).

3.4.3. What is the recommended type and intensity level of exercise for losing weight or body fat and maintaining muscle mass?

Aerobic and strength exercises can lead to weight loss, body fat reduction, and muscle mass loss in patients with obesity (22-26). The amount of weight loss and fat loss resulting from aerobic exercise is greater than that achieved through strength exercise (3 kg compared to 1 kg). In contrast, loss of muscle mass during weight loss can be better prevented through strength exercises than through aerobic exercises (27).

It is recommended to do approximately 300 minutes of moderate aerobic exercise or 150 minutes of combined vigorous and moderate exercise per week, as well as calorie intake reduction (A). It is preferable to begin with 30 to 60 minutes of moderate-intensity aerobic exercise daily before gradually increasing the duration to 90 minutes (A). It is recommended to do strength training with exercise Thera-bands, dumbbells, or weight machines 2 - 3 sessions a week. Each session should include 2 - 4 sets of each exercise repeated 8 to 12 times and should be 60 to 70% of one-repetition maximum (1RM) (A). Both types of training should start based on an individual’s baseline fitness level and then gradually increase in intensity and duration. It should also be kept in mind that strength training may initially lead to weight gain due to increases in muscle mass. In addition, stretching exercises should be performed on most days of the week to maintain the range of motion of the joints, muscles, and tendons around the joints. Each stretching exercise should last for 10 to 30 seconds and be repeated at least four times per session (A).

In settings with limited access to advanced monitoring tools, exercise intensity may be estimated using clinical methods such as perceived exertion and functional capacity assessment.

It should be noted that exercise intensity and progression should be individualized based on baseline fitness level, comorbidities, and clinical status, and should follow appropriate medical screening recommendations as outlined in Recommendation 6.

#### 3.4.4. Can Exercise Before and After MBS Improve Cardiorespiratory Fitness and Physical Function?

Both aerobic and strength exercises can improve cardiorespiratory fitness in people with obesity (24, 27-35). Many studies have corroborated the positive effect of physical activity on strength and physical function in the postoperative period (16, 36-50).

Due to the paucity of long-term studies, it is not possible to conclude whether the effect of exercise in the preoperative period persists following surgery or whether exercise in the postoperative period is responsible for this improvement (51). However, based on the proven positive effects of exercise on functional fitness at each stage (pre- or post-MBS), it is recommended to do aerobic, strength, and flexibility exercises, or a combination thereof, before and after surgery to increase cardiorespiratory and physical fitness (A).

#### 3.4.5. Can Exercise Before Surgery Increase the Amount of Physical Activity After Surgery?

Most candidates for MBS are less physically active than healthy lean individuals. Fewer than 5% of MBS candidates meet the physical activity guidelines of the American College of Sports Medicine (ACSM) (52). Many continue to be physically inactive after MBS (16, 52-54). Research shows that 90% of people are insufficiently active in the postoperative period according to international guidelines (55).

Several studies report higher physical activity after surgery than in the preoperative period (16, 17, 56, 57). This may be a consequence of weight loss resulting from surgery or a high level of physical activity before surgery. Indeed, higher levels of physical activity before MBS can predict higher levels of physical activity after MBS (16, 36, 54, 58).

Despite the limited scientific evidence regarding the effect of preoperative physical activity on postoperative physical activity, it is generally understood that individuals who are physically active before MBS will continue to be more active after MBS. Hence, it is recommended that people with obesity engage in as much physical activity as possible before MBS to ensure greater activity in the postoperative period (B).

#### 3.4.6. Is There a Need for Medical Evaluation Before Starting Exercise in People with Obesity?

Performing low-impact or moderate exercise usually does not require a specific medical examination. However, as patients with obesity may have other health issues such as HTN, T2D, DLP, and CVD, the ACSM guidelines recommend that individuals with obesity and cardiovascular, renal, or metabolic diseases start with light to moderate exercise without a medical assessment.

If a patient with obesity demonstrates signs and symptoms of cardiovascular conditions, renal disease, or diabetes, medical supervision is required even before engaging in low-intensity exercise (A). A medical evaluation may involve taking a medical history and performing a physical examination, electrocardiography, cardiac stress testing, and other investigations. If a patient with obesity has lower-body musculoskeletal issues and cannot walk on a treadmill, cardiac stress testing may be performed using a stationary exercise bike or an upper-body ergometer. Some conditions such as ongoing cardiovascular diseases, uncontrolled hypertension, physical disabilities, deep vein thrombosis, and pulmonary infarction are considered contraindications to exercise.

#### 3.4.7. What Types and Intensity Levels of Exercise After MBS Can Help with Weight Loss and Fat Reduction?

Studies show that individuals with a regular exercise program after MBS achieve greater weight loss compared with those without (27, 28, 59-62). It is recommended to follow a regular regimen combining aerobic and strength training after surgery to achieve greater weight loss (A). It is preferable to inform patients that average weight loss with postoperative exercise is not more than 2 - 3 kg (B). In general, it is recommended that individuals engage in moderate postoperative exercise for at least 30 minutes per day, five times a week, totaling 150 minutes per week. However, for additional weight loss, it is advisable to increase the duration to 60 minutes per day, at least five days per week, amounting to 300 minutes per week. Each exercise session should incorporate a warm-up, aerobic activity, stretching, strength training, and a cool down period (B). Patients who participate in supervised low-intensity strength exercise for 24 weeks achieve significant reductions in body weight (63-67). Those who engage in more than 150 minutes of moderate-intensity physical activity per week achieve greater weight loss and fat loss 6 and 12 months after surgery compared with individuals who take part in less than 150 minutes of physical activity. It is recommended that individuals who have undergone bariatric surgery engage in at least 150 to 200 minutes of moderate-intensity aerobic physical activity each week (A).

It should be noted that exercise intensity and progression should be individualized based on baseline fitness level, comorbidities, and clinical status, and should follow appropriate medical screening recommendations as outlined in Recommendation 6. Higher-intensity targets should be intended primarily for clinically stable patients and for those who have received appropriate medical clearance.

#### 3.4.8. What Is the Best Prescription for an Aerobic Exercise Regimen After MBS?

Aerobic exercise can be initiated in the second week following MBS. Typically, during the third and fourth weeks, it is recommended to exercise at an intensity of 50% of Heart Rate Reserve (HRR) with a frequency of three times per week, with each session lasting 30 minutes. The first and last five minutes of each exercise session should be dedicated to warming up and cooling down, respectively. However, this should be tailored to the individual’s fitness level. From the fourth to eighth weeks, aerobic exercise should be performed three to four times a week at an intensity of 60% to 70% of HRR, with each session lasting 40 minutes (68). In the ninth to twelfth weeks, it is generally advisable to exercise five times a week at an intensity of 70% to 80% of HRR for 60 minutes per session (A).

HRR can be estimated using age-predicted HRmax (220-age) and resting HR. Formal cardiopulmonary exercise testing is also recommended when available but is not mandatory for all patients.

It should be noted that exercise intensity and progression should be individualized based on baseline fitness level, comorbidities, and clinical status, and should follow appropriate medical screening recommendations as outlined in Recommendation 6. Progression to higher-intensity aerobic exercise (e.g., ≥70% HRR) should be considered only after appropriate clinical evaluation and in clinically stable patients, particularly in those with cardiometabolic comorbidities or low baseline fitness.

#### 3.4.9. What Is the Best Prescription for a Strength Exercise Regimen After MBS?

To maintain muscle mass following MBS-induced weight loss, it is advisable to engage in moderate to vigorous strength training or a combination of strength and aerobic exercises (27, 69, 70). Strength training should target the major muscle groups of the body, including the trunk, lower limbs, shoulders, and arms. It is recommended to begin strength training two weeks after MBS. Typically, this training is performed during the third and fourth weeks at an intensity of 50 to 60% of one-repetition maximum (1RM), with a frequency of twice a week. The first and last five minutes of each exercise session should be dedicated to warming up and cooling down, in that order, combining light aerobic activities and stretching exercises. From weeks four to eight, strength training should be performed three times a week at an intensity of 60 to 70% of 1RM, with each session lasting approximately 40 minutes. During weeks nine to twelve, it is generally recommended to extend each session to 60 minutes at an intensity of 50 to 75% of 1RM. To effectively maintain weight loss, it is essential to continue the exercise regime for a minimum of 12 weeks, preferably extending to at least nine months or a year. However, it is important to note that the initiation and continuation of strength training should be tailored to each individual’s baseline physical conditions, as these factors can vary significantly between individuals (A).

It should be noted that exercise intensity and progression should be individualized based on baseline fitness level, comorbidities, and clinical status, and should follow appropriate medical screening recommendations as outlined in Recommendation 6. In sedentary individuals, 10 RM estimation is used rather than formal 1RM testing.

#### 3.4.10. Is It Possible to Have a Similar Recommendation for Physical Activity for All People with Obesity of All Ages?

In people with obesity or overweight aged more than 40 years, health status should be considered before starting exercise or increasing intensity. It is recommended that a physical examination and, if necessary, cardiovascular and laboratory evaluation be performed and a full medical history be taken before the individual engages in physical activity (D). Patients with obesity who lead very sedentary lives are advised to start with 5 to 10 minutes of exercise per day and gradually increase intensity. Alternatively, instead of one session per day, they can start physical activity with short 10-minute sessions several times a day and gradually raise the bar (D).

#### 3.4.11. Does Regular Exercise Increase Insulin Sensitivity in Patients with Obesity and Diabetes?

In patients with obesity with or without diabetes, regular moderate aerobic exercise improves insulin sensitivity and cellular glucose utilization, as well as promoting weight loss and improving cardiorespiratory fitness (27). It is recommended that all people with overweight or obesity, regardless of MBS, engage in regular exercise (aerobic, strength, or a combination thereof) to increase insulin sensitivity (A).

#### 3.4.12. Does Exercise After MBS Lead to Greater Weight Loss and Fat Loss?

Studies have shown that exercise after MBS can lead to greater weight loss and fat loss (28, 61, 62, 71-73). Although few studies have examined the long-term effects of exercise, several studies on the durability of exercise effects have shown that weight loss is maintained in the long run (i.e., up to 24 months) (57, 60, 61). Therefore, it is recommended that all patients undergoing various MBS procedures have a regular exercise program in the postoperative period and try to continue exercising for a longer period (A).

#### 3.4.13. Does Exercise After MBS Prevent Recurrent Weight Gain?

Research indicates that regular physical activity can help maintain weight loss for an extended duration after MBS and prevent weight regain (61, 74-81). The most significant weight loss typically occurs between 12 and 24 months following surgery, with the percentage of weight loss generally decreasing after this period. Engaging in moderate exercise over this timespan can help sustain the weight loss achieved after surgery. Consequently, all patients are encouraged to adhere to an exercise program that consistently incorporates both aerobic and strength training over the long term to mitigate the risk of recurrent weight gain (A).

#### 3.4.14. Does Exercise in the Postoperative Period of MBS Improve Cardiorespiratory Fitness Indices?

Major changes in cardiorespiratory fitness indices occur after surgery, and exercise can enhance the rate of improvement in these indices (24, 61, 82-84), with effects lasting up to 24 months (83). All patients with obesity are advised to maintain a regular, long-term exercise regimen in the postoperative period to maximize improvements in cardiorespiratory fitness (A).

#### 3.4.15. Does Exercise Improve Muscle Strength and Reduce Muscle Tissue Loss After MBS?

After MBS, fat-free mass, which includes muscle tissue, decreases significantly in all parts of the body along with fat mass reduction (85-91). The greatest amount of weight loss occurs during the first quarter after MBS (87). Muscle mass reduction can severely affect a person’s quality of life and may even lead to increased appetite and weight gain in the postoperative period due to changes in energy balance. In general, muscle mass reduction may threaten health in the long run (92-97). In the postoperative period, exercise can improve muscular fitness in addition to increasing weight loss and improving cardiorespiratory fitness (3, 24, 27, 61, 72, 73, 98-101). Although the positive effect of strength training on muscle fitness and strength has been well substantiated, several studies show that this effect decreases after exercise cessation and does not persist for long (61, 72, 73, 102). The effect of exercise has been observed to last for a maximum of 3 - 12 months whether or not strength training is performed during the five-year follow-up (103). Therefore, it is recommended that regular strength training be included in the daily routine after MBS in the long term to minimize the loss of lean mass and improve muscle strength (B). Strength training, alone or in combination with aerobic exercise, can improve muscle strength in people with obesity more than aerobic exercise alone (A).

#### 3.4.16. Is There a Precise Method for Measuring the Amount of Physical Activity Done After MBS?

There is no gold standard for assessing physical activity in patients who have undergone bariatric surgery. However, software installed on smartwatches, smartphones, or pedometers can be used, along with less precise questionnaires, to assess physical activity (D).

Table 1 presents the evidence mapping for all recommendations, allowing transparent visualization of the quantity and quality of evidence supporting each statement. The table demonstrates that most recommendations are supported by multiple clinical studies with acceptable methodological quality, whereas a smaller number are based on limited direct evidence and were supplemented by expert consensus through the Delphi process. The variability in risk of bias mainly reflects differences in study design and reporting quality across the available literature.

## 4. Discussion

MBS is a widely accepted procedure for managing obesity and its associated comorbidities. However, practical clinical guidelines that provide detailed instructions regarding exercise before and after MBS remain lacking.

This guidance provides the most recent evidence on exercise prescriptions for individuals undergoing MBS. It highlights the vital role of structured physical activity, including enhanced weight loss outcomes, preservation of lean mass, maintenance of reduced weight, improved metabolic health, and better postoperative outcomes. Due to the absence of specific exercise guidelines for this patient population, much of the evidence supporting these recommendations was derived from meta-analyses, systematic reviews, high-quality randomized controlled trials (RCTs), and general guidelines on obesity treatment. However, some recommendations lack direct evidence because of the limited number of studies addressing specific questions; therefore, expert opinion played a key role.

Preoperative exercise may contribute to greater weight loss following MBS (18-20, 54, 58). Although the scientific evidence supporting this is limited, the guidance recommends that patients begin exercising at least three months before MBS to foster behavioral changes that facilitate postoperative adherence. Research indicates that aerobic exercise, even in isolation, can reduce abdominal visceral fat and overall body weight (27). Increasing muscle mass and strength is essential for restoring physical function and improving quality of life, and these benefits can be sustained after MBS (27-51). Accordingly, incorporating resistance exercise alongside aerobic activity before MBS is recommended. Nonetheless, further research is needed to evaluate the specifics of strength training, including variations in exercise intensity and frequency, to optimize pre- and post-MBS outcomes (104).

Postoperatively, aerobic exercise not only promotes greater weight and fat loss but also reduces the likelihood of weight regain and improves lean mass status (61, 72, 73, 98-101, 105). Therefore, combining aerobic and resistance exercise is recommended as the optimal exercise prescription following MBS.

In many existing studies on exercise therapy and MBS, the details of exercise protocols have not been thoroughly described. Therefore, this guidance presents the most practical and effective protocol for aerobic and resistance exercise before and after MBS by integrating the panel members' expertise with the available evidence.

### 4.1. Strengths and Limitations

The most significant strength of this study is that it introduced a global evidence-based practice approach focused on exercise therapy in MBS. Another key strength is its use of the three-round DELPHI method and the AGREE checklist, ensuring a systematic and evidence-based approach.

One limitation of this study is the lack of a guideline specifically focused on exercise in MBS. Furthermore, when addressing certain clinical questions (e.g., questions 2, 10, and 16 in Appendix 2 in the Supplementary File), the available evidence and number of studies were extremely limited, resulting in reliance on the expertise and experience of the panel members. Thus, reliance on expert consensus could limit generalizability and reduce the strength of the evidence. Additionally, most studies primarily addressed the short-term postoperative period, leaving gaps in long-term outcomes. Other limitations of this guideline include limited direct evidence for specific subgroups (age-specific adaptations, body composition outcomes), the lack of formal patient participation in the Delphi voting rounds, and potential regional applicability (e.g., applicability to non-Iranian or non-Middle Eastern populations).

### 4.2. Conclusion

Overall, this guidance recommends exercise both before and after MBS to improve quality of life, reduce the probability of weight regain, and control complications and associated medical problems attendant upon obesity. It also highlights the need for future empirical validation of the proposed exercise recommendations through meticulous controlled trials.

ijem-24-3-166557-s001.pdf
