# Supplementary material for: Development of an Iranian Clinical Guideline on Perioperative Exercise Therapy for Metabolic and Bariatric Surgery: A Delphi Consensus Study Using the AGREE II Framework
Source: Int J Endocrinol Metab. 2026 May 25;24(3):e166557. doi: 10.5812/ijem-166557 (PMC13402807; doi:10.5812/ijem-166557)
Supplement: ijem-24-3-166557-s001.pdf [file ijem-24-3-166557-s001.pdf]

Appendix 1.

1. Canadian Adult Obesity Clinical Practice Guidelines, https://obesitycanada.ca/healthcare-professionals/adult-clinical-practice-guideline/

2. Wharton S, Lau DCW, Vallis M. and et al. Obesity in adults: a clinical practice guideline. CMAJ. 2020;192(31): E875-E891. doi: 10.1503/cmaj.191707. PMID: 32753461; PMCID: PMC7828878

3. Coen PM, Carnero EA, Goodpaster BH. Exercise and Bariatric Surgery: An Effective Therapeutic Strategy. Exerc Sport Sci Rev. 2018 Oct;46(4):262-270. doi: 10.1249/JES.0000000000000168. PMID: 30052546; PMCID: PMC6147093.

4. Obesity: identification, assessment and management. Published 2014, Last updated 2023. https://www.nice.org.uk/guidance/cg189.

5. Weight management: lifestyle services for overweight or obese adults, Public health guideline Published 2014. https://www.nice.org.uk/guidance/ph53.

6. Eduardo Grunvald, Raj Shah, Ruben Hernaez, et al. AGA Clinical Practice Guideline on Pharmacological Interventions for Adults with Obesity. Gastroenterology 2022; 163:1198–1225.Guidelines

7. Eisenberg D, Shikora SA, Aarts E, et al. 2022 American Society of Metabolic and Bariatric Surgery (ASMBS) and International Federation for the Surgery of Obesity and Metabolic Disorders (IFSO) Indications for Metabolic and Bariatric Surgery. Obes Surg. 2023 Jan;33(1):3-8. Erratum in: Obes Surg. 2022; PMID: 36336720; PMCID: PMC9834364. doi: 10.1007/s11695-022-06332-1.

9. ASMBS_IFSO Guidelines on indications for metabolic and bariatric surgery, 2022.

10. Elrazek AE, Elbanna AE, Bilasy SE. Medical management of patients after bariatric surgery: Principles and guidelines. World J Gastrointest Surg.2014 Nov 27;6(11):220-8. doi: 10.4240/wigs. v6. i11.220. PMID: 25429323; PMCID: PMC4241489.

11. Martin Fried; Volkan Yumuk; Jean-Michel Oppert , et al. Interdisciplinary European Guidelines on Metabolic and Bariatric Surgery. Obes Facts, 2013; 6 (5): 449–468. https://doi.org/10.1159/000355480

12. Mary O'Kane, Helen M Parretti, Carly A Hughes, et al. Guidelines for the follow-up of patients undergoing bariatric surgery. Clinical Obesity, 2016 https://doi.org/10.1111/cob.12145

13. Melissas, J. IFSO Guidelines for Safety, Quality, and Excellence in Bariatric Surgery. OBES SURG 18, 497–500 (2008). https://doi.org/10.1007/s11695-007-9375-9

14. Walter J. Pories, Lynis G. Dohm, Christopher J. Mansfield Beyond the BMI: The Search for Better Guidelines for Bariatric Surgery. Obesity a research journal. 2012 https://doi.org/10.1038/oby.2010.8

15. Janey S.A. Pratt, Allen Browne, Nancy T, et al. ASMBS pediatric metabolic and bariatric surgery guidelines, 2018, Surgery for Obesity and Relate Diseases. Journal and books 2018; 14(7), 882-901. ISSN 1550-7289, https://doi.org/10.1016/j.soard.2018.03.019. https://www.sciencedirect.com/science/article/pii/S155072891830145X

16. Runkel N, Colombo-Benkmann M, et al. Bariatric surgery. Dtsch Arztebl Int, 2011;108(20):341-6. doi: 10.3238/arztebl.2011.0341. PMID: 21655459; PMCID: PMC3109275.

17. Hussien, J., Asselin, M., Bond, D., Wu, Y., Ly, V., Creel, D., Papasavas, P., Goodpaster, B. H., & Baillot, A. (2025). Exercise training in metabolic and bariatric surgery: An overview of systematic reviews. Obesity Reviews, e13920-. https://doi.org/10.1111/obr.13920

18. Cornier M A. A Review of Current Guidelines for the Treatment of Obesity. Am J Manag Care. 2022;28(suppl 15): S288-S296. doi:10.37765/ajmc.2022.89292

19.HAMPL, Sarah E., et al. Clinical practice guideline for the evaluation and treatment of children and adolescents with obesity. Pediatrics, 2023, 151.2.

Appendix 2.

1. Does exercise before MBS lead to more significant weight loss after MBS?
2. Can exercise before MBS reduce body fat percentage after MBS?
3. What is the recommended type and intensity level of exercise for losing weight or body fat and maintaining muscle mass?
4. Can exercise before and after MBS improve cardiorespiratory fitness and physical function?
5. Can exercise before MBS increase the amount of physical activity after MBS?
6. Is there a need for medical evaluation before starting exercise in individuals with obesity?
7. What type and intensity level of exercise after MBS can help with weight loss and fat reduction?
8. What is the best prescription for an aerobic exercise regimen after MBS?
9. What is the best prescription for a strength exercise regimen after MBS?
10. Is it possible to have a similar recommendation for physical activity for all people with obesity in all group ages?
11. Does regular exercise increase insulin sensitivity in patients with obesity and diabetes?
12. Does exercise after MBS lead to greater weight loss and fat loss?
13. Does exercise after MBS help prevent regaining weight?
14. Does exercise in the postoperative period of MBS improve cardiorespiratory fitness indices?
15. Does exercise improve muscle strength and reduce muscle tissue loss after MBS?
16. Is there a precise method for measuring the amount of physical activity done after MBS?

Appendix 3.

Forms 1-3

Form 1: Developing the Clinical Questions

Form 2: Clinical Benefits of the Clinical Scenarios

Form 3: Scoring the Scenarios

- Scottish Intercollegiate Guidelines Network (SIGN): www.sign.ac.uk
- National Health and Medical Research Council (NHMRC): www.nhmrc.gov.au
- National Guidelines Clearinghouse (NGC): www.guideline.gov
- Guidelines Advisory Committee (GAC): www.gacguidelines.ca
- New Zealand Guidelines Group: www.health.govt.nz/publications
- National Institute for Clinical Evidence (NICE): www.nice.org.uk

Form 1

**Providing the Recomendations**		**Diagnostic or Therapeutic**	Clinical Question (based on PICO)
**Recommendation**	**Guideline**		
**In patients scheduled to receive gastric bypass surgery, participation in pre-operative weight loss programs does not lead to greater post-surgical weight loss.** 2+	Scottish Intercollegiate guideline(١١۵,Management of Obesity A national clinical guideline)	**T**	Does performing exercise before surgery in patients who are candidates for bariatric surgery influence the amount of weight loss after the operation?

**For adults who are overweight or obese, prescribe approximately 300 minutes of moderate-intensity activity, or 150 minutes of vigorous activity, or an equivalent combination of moderate-intensity and vigorous activities each week combined with reduced dietary intake.**<br><br>**(CBR)Consensus-based recommendation formulated in the absence of quality evidence**<br><br>**Suggest long-term, regular physical activity—30 to 60 minutes daily at moderate intensity— to all overweight and obese patients. [Level of Evidence: 2, Grade: B]**	summary guide for the management of overweight and obesity in primary care/ December 2013<br><br>www.gacguidelines.ca (The Guidelines Advisory Committee)	T	Before undergoing surgery, what intensity of exercise is recommended for weight loss?
**Recommend endurance training for adults. [Level of Evidence: 2, Grade: B] . Suggest that patients gradually increase the total duration of the exercise. [Level of Evidence: 2, Grade: A]**<br><br>**Assess patients before they embark on a vigorous exercise program. [Level of Evidence: 4, Grade: C]**<br><br>**Exercise may consist of aerobic activity, flexibility-based activity and /or anaerobic activity such as**	www.gacguidelines.ca (The Guidelines Advisory Committee)<br><br>Clinical Guidelines for Weight Management in New Zealand	T	What type of exercise do you recommend for weight loss before surgery?

**weight training (Ainsworth et al., 2011).**<br>**Include muscle strengthening/resistance activities on two or more days of the week. Note that muscle-strengthening activities may result in initial weight gain, due to changes in muscle mass.**	Adults (Ministry of Health 2017)<br>Australian Government/ National health and medical research council		
**The studies found a significant decrease in body weight, BMI, fat mass after a 24-week supervised low-intensity endurance training. (PP**<br>**(Developed by the Obesity Guidelines Development Committee for areas beyond the scope of the systematic review)**	Scottish Intercollegiate guideline<br>(Management of Obesity, A national clinical guideline)	T	Does combining supervised aerobic and resistance exercise improve anthropometric factors in patients who are candidates for bariatric surgery
**In one cohort study, patients who had had bariatric surgery and reported >150 minutes moderate physical activity per week had greater excess weight loss at six and 12 months post-surgery than those taking <150 minutes per week.**	National Health and Medical Research Council (2013), Clinical practice guidelines for the management of overweight and obesity in adults, adolescents and children in Australia.	T	Does exercise intensity in the postoperative period after bariatric surgery affect the amount of weight loss?
**For adults who are overweight or obese, particularly those who are older than 40 years,**	National Health and Medical Research Council	T	

**there should be an individualized approach to increasing physical activity.**			Can a single physical activity recommendation be applied to all obese individuals at any age?
**If you are medically stable but have not recently been regularly active, you may wish to:**<br>**– start with 5 or 10 minutes of exercise a day and work up to more**<br>**– split physical activity into smaller bouts (eg, walking for 10 minutes after each meal) to reach a daily activity target, instead of undertaking it all at once.**	Clinical Guidelines for Weight Management in New Zealand Adults (Ministry of Health 2017)	**T**	What recommendations do we give for starting exercise in individuals who previously had a sedentary lifestyle?
Results from a larger randomized controlled trial indicate that moderate aerobic exercise elicits additional improvements in insulin sensitivity and glucose effectiveness, i.e., the ability of glucose per se to facilitate glucose disposal, along with improved cardiorespiratory fitness during RYGB surgery-induced weight loss	National Health and Medical Research Council (2013), Clinical practice guidelines for the management of overweight and obesity in adults, adolescents and children in Australia. National Health	**T**	Is regular exercise effective in increasing insulin sensitivity in individuals with diabetes?

There was a significant reduction in weight and BMI in training group and control group 4 months after bariatric surgery compared to the preoperative values. It is known that exercise results in greater weight loss than bariatric surgery alone.	and Medical Research Council<br><br>DOI: 10.1159/000381201	**T**	Does exercise in the postoperative period after bariatric surgery lead to greater ?weight loss
Data from the National Weight Control Registry (NWCR) and from other investigations suggest that moderate intensity exercise is critical for maintaining weight loss<br><br>Physical activity causes the long term maintenance of weight loss in bariatric surgery.<br><br>II-1 , 2B	National Health and Medical Research Council	**T**	Does exercising after bariatric surgery prevent weight regain

There are significant changes of anthropometric parameters following bariatric surgery.	DOI: 10.1159/000381201	**T**	Does exercise in the post-bariatric surgery period lead to greater improvement in ?anthropometric factors
Exercise training can attenuate muscle atrophy and can maintain FFML(Fat Free Mass Loss) during weight loss [36, 37] ,<br><br>To date, the published data support a potential role for exercise to elicit positive changes in body composition following bariatric surgery<br><br>This evidence suggests that exercise is feasible for bariatric surgery patients and provides improvements in fitness and muscle strength in addition to the benefits of bariatric surgery-induced weight loss, data that strongly advocate for the inclusion of an exercise program to optimize health benefits during active weight loss following bariatric surgery	DOI: 10.1159/000381201<br><br>And<br><br>National Health and Medical Research Council	**T**	Does exercise during the post-surgery period reduce muscle mass loss?"

Postoperative exercise is associated with greater weight loss at 12 and 24 months after bariatric surgery. The long-term benefit of exercise in this patient population remains to be shown	National Health and Medical Research Council	**T**	For how long after surgery does regular exercise contribute to weight loss?
There is no single instrument that is accepted as the gold standard for measuring exercise in bariatric patients	National Health and Medical Research Council	**D**	What is the accurate method for measuring the amount of exercise ?performed after bariatric surgery

For weight maintenance, do 150 to 250 minutes of moderate-intensity aerobic physical activity spread over the week (e.g., 30 minutes a day, five days a week).	Clinical Guidelines for Weight Management in New Zealand Adults (Ministry of Health 2017)	**T**	In someone who has lost weight after surgery, how much physical activity is necessary to maintain weight?

Form 2

Side effects	Effectiveness	Benefits	Cost	Evidence	Scenarios	Clinical Issue
Inconclusive		The amount of weight regain is lower than in the group that did not exercise.		An individual's BMI and lifestyle before surgery affect weight regain after surgery	Does preoperative exercise affect the amount of weight regain ?after surgery	Does performing exercise before surgery in patients who are candidates for bariatric surgery influence the amount of weight loss after the operation?
	mean of 4.4 to 21.0 minutes/day), VS (mean of 7.9 to 7.6 minutes/day)	• 147 minutes/ week of physical activity<br>• Improved quality of life and better feelings during physical activities<br>• Improved physical fitness<br>• Improved		Postoperative physical activity levels in the group that was active before surgery are five times higher than in those who only received preoperative care	Preoperative exercise can influence the level of physical activity after surgery.	

		6-minute walk test performance<br><br>• Improved muscle strength				
		6MWT, sit to stand, half squat, arm curl		Twelve weeks of combined aerobic and resistance exercise after surgery, compared to a control group, led to improvements in walking distance (three sessions per week of moderate-intensity aerobic exercise combined with 60 minutes of resistance training)	Aerobic and resistance exercises can improve an individual's functional capacity after surgery.	How much does post-surgery exercise affect weight loss and functional capacity?
	- 52.5 +/- 15.4 vs. 46.4 +/- 12.8 kg) and BMI (18.9 +/-			Individuals who engaged in more than 200 minutes of	Exercise during the post-surgery period is associated with greater weight loss	

	4.6 vs. 16.9 +/- 4.2 kg/m(2)):P< 0.01			physical activity per week experienced greater weight loss compared to those with less than 200 minutes.		
	(95% CI = -0.02 to 0.32)<br><br>P.V= 0.094			A systematic review showed that exercise during the post-surgery period has no effect on weight loss.	Exercise during this period does not lead to greater weight loss.	
	8.1±5.0 kg (9.3±5.6%) at 6 months, 7.8±6.8 kg (8.9±7.8%) at 12 months, 5.9±7.0 kg (6.8±8.1%) at 18 months, and 4.2±7.2 kg (5.0±8.5%) at 24 months.<br><br>P.V > 0.05	هر شدتی از ورزش در دوران پس از جراحی توسط افراد قابل تحمل است و تفاوتی بین اثر آنها وجود ندارد		Moderate physical activity (~1000 kcal/week) and vigorous physical activity (~2000 kcal/week) did not differ in terms of weight loss after 6 months and 2 years.	The intensity and duration of exercise in the post-surgery period do not affect the amount of weight loss.	Do resistance exercises alongside aerobic exercises affect the pattern of weight loss?
	group-by- + week	High-volume physical		Physical activity that	High-intensity exercise after bariatric surgery	

	effect: P = 0.009-0.03	activity is tolerable by 50% of patients and has a greater positive effect on step count and VO_2max, although there is no difference in blood glucose and lipid levels between the two groups		expends more than 2000 kilocalories per week during the post-bariatric surgery period has a greater positive effect on anthropometric factors and postprandial blood glucose compared to the control group	has a greater positive effect on improving anthropometric factors.	
	Helps increase basal metabolic rate while preserving lean body mass	• Potential joint injury if using weights beyond one's tolerance."<br><br>• Increased cardiovascular strain if unaware of proper breathing techniques during resistance exercises."<br><br>• Increased	5 million Rials / month	CON: mean,−8.8 kg; 95% CI: −10.1 to −7.5 kg; PRO: mean, −8.2 kg; 95% CI: −9.3 to −7.1 kg; PRO+EX: mean, −7.7 kg; 95% CI: −9.0 to −6.5 kg; *P* = 0.899).	Resistance Training and Protein Supplementation Increase Strength After Bariatric Surgery: A Randomized Controlled Trial doi.org/10.1002/oby.22317<br><br>Effectiveness of exercise training after bariatric surgery —a systematic literature review and meta-analysis 10.1111/obr.12740<br><br>Micronutrient and protein deficiencies after gastric bypass and sleeve gastrectomy: a 1-year follow-up	Engaging in resistance exercises for at least 6 months, along with adequate protein intake, helps preserve body muscle mass."

		load on the renal system if protein supplements are overused and fluid intake is inadequate			10.1007/s11695-015-1803-7	
	It helps improve mobility, particularly walking, in post-surgical patients, which contributes to weight loss and enhanced quality of life	Potential joint injury if using weights beyond one's tolerance."<br><br>● Increased cardiovascular strain if unaware of proper breathing techniques during resistance exercises."<br><br>● Increased load on the renal system if protein supplements are overused and fluid intake is inadequate	5 million Rials / month	PRO+EX group (+0.6 [0.3 to 0.8]) versus +0.1 (−0.1 to 0.4) and +0.2 (0.0 to 0.4) kg/kg body mass in CON and PRO groups, respectively ($P = 0.021$).	Resistance Training and Protein Supplementation Increase Strength After Bariatric Surgery: A Randomized Controlled Trial doi.org/10.1002/oby.22317<br><br>Effect of a randomized 12-week resistance training program on muscular strength, crosssectional area and muscle quality in women having undergone Roux-en-Y gastric bypass 10.1080/02640414.2017.1322217	Resistance exercises alongside aerobic exercises have an effect on improving individual performance."<br><br>Performing resistance exercises for at least 6 months, along with adequate protein intake, increases muscle strength, particularly in the lower limbs

	The likelihood of developing or progressing metabolic diseases decreases, and the patient's physical fitness will also improve.	If exercise is performed irregularly, optimal blood glucose control may not be achievable. It is necessary to perform aerobic and resistance exercises with a specific frequency to achieve better glycemic control.		S_I improved in both groups following the intervention (ITT: CON vs. EX; +1.64 vs. +2.24 $min^{-1}/\mu U/ml$, *P* = 0.18 for Δ, *P* < 0.001 for time effect).<br><br>S_I improvement (PP: CON vs. EX; +1.57 vs. +2.69 $min^{-1}/\mu U/ml$, *P* = 0.019) above that of surgery.<br><br>S_G (ITT: CON vs. EX; +0.0023 vs. +0.0063 min^{-1}, *P* = 0.009) compared with the CON group	Clinical trial demonstrates exercise following bariatric surgery improves insulin sensitivity 10.1172/JCI78016	The effect of regular exercise after bariatric surgery on cardiometabolic factors, independent of the surgical effect, is unclear.<br><br>The impact of exercise on insulin sensitivity and glycemic control, independent of the bariatric surgery process, is uncertain.<br><br>However, it appears that individuals who engage in regular exercise after bariatric surgery exhibit better insulin sensitivity and glycemic control

						compared to sedentary individuals, even with similar amounts of weight loss.

Form 3

1-9	Scoring form								
	Adaptability			Clinical benefits			Scenarios	Clinical Questions	
	Low	Intermediate	High	Low	Intermediate	High			
5		■		■			Preoperative exercise has a positive effect on weight regain after surgery.	1	Does performing exercise before surgery in patients who are candidates for bariatric surgery influence the amount of weight loss after the operation
9			■			■	Preoperative exercise positively affects physical activity levels after surgery	2	
7		■			■		The type of exercise (combination of aerobic and resistance training) is effective in improving an individual's functional capacity after surgery	3	
7		■				■	Exercise, compared to no exercise before surgery, is associated with greater weight loss	۴	
9			■			■	Aerobic and resistance exercises can improve an individual's functional performance after surgery	1	How much does post-surgery exercise affect weight loss and an individual's functional capacity?

9			■			■	Exercise during the post-surgery period increases the amount of weight loss.	٢	
1	■			■			Exercise during the post-surgery period does not lead to greater weight loss.	3	
8			■			■	Regular and supervised exercise is associated with greater weight loss.	4	
9			■			■	Regular and supervised exercise is associated with greater improvement in performance	5	
1	■			■			Exercise intensity and duration in the post-surgery period do not affect the amount of weight loss	6	
8			■			■	High-intensity exercise after bariatric surgery has a greater positive effect on improving anthropometric factors	7	
7		■			■		Resistance exercises alongside aerobic exercises are associated with greater weight loss.<br>Performing resistance exercises for at least 6 months, along with adequate protein intake, helps preserve lean body mass	8	
5		■			■		The effect of different types of exercise on preserving lean body mass after surgery is unclear	9	

7		■				■	Resistance exercises alongside aerobic exercises have an effect on improving an individual’s functional performance.<br>Performing resistance exercises for at least 6 months, along with adequate protein intake, increases muscle strength, particularly in the lower limbs	10	
7		■				■	The effect of exercise on insulin sensitivity and glycemic control, independent of the bariatric surgery process, is unclear. However, it appears that individuals who engage in regular exercise after bariatric surgery have better insulin sensitivity and glycemic control compared to sedentary individuals, even with similar amounts of weight loss	١١	The effect of regular exercise after bariatric surgery on cardiometabolic factors, independent of the surgical effect, is unclear
